# Beyond a binary RTK-RAS biomarker: context-aware precision oncology in early-onset colorectal cancer

**DOI:** 10.46439/cancerbiology.7.085

**Published:** 2026

**Authors:** Enrique Velazquez-Villarreal

**Affiliations:** 1 City of Hope, Beckman Research Institute, Department of Integrative Translational Sciences, Duarte, CA, USA; 2 City of Hope Comprehensive Cancer Center, Duarte, CA, USA

**Keywords:** Colorectal cancer, Early-onset colorectal cancer, RTK–RAS pathway, FOLFOX, Artificial intelligence, AI-agents

## Abstract

Early-onset colorectal cancer (EOCRC) is increasing worldwide and represents a biologically and clinically heterogeneous disease that requires precision approaches extending beyond conventional single-gene biomarkers. Recent analyses of receptor tyrosine kinase-RAS (RTK-RAS) pathway alterations demonstrate that their clinical significance is strongly dependent on age at diagnosis, ancestry, and treatment exposure. In particular, RTK-RAS alterations have been associated with unfavorable survival in untreated early-onset non-Hispanic White colorectal cancer, while an opposite association has been observed in FOLFOX-treated late-onset disease. At the same time, overall RTK-RAS pathway alteration frequencies remain relatively stable across patient groups, whereas gene-specific differences involving ERBB2, NF1, FGFR2, NTRK2, and other pathway components reveal substantial molecular heterogeneity. These observations challenge the use of RTK-RAS alteration status as a uniform prognostic biomarker and instead support a context-dependent model in which genomic alterations interact with age-related tumor biology, treatment pressures, ancestry-associated factors, and broader molecular states. Emerging genomic studies further suggest that EOCRC may harbor distinct mutational processes related to earlier-life exposures, reinforcing the need to integrate pathway alterations with mutational signatures, tumor microenvironment features, and multi-omic information. Artificial intelligence-enabled platforms such as AI-HOPE, developed at the Velazquez-Villarreal Lab at City of Hope, provide a scalable strategy for interrogating these complex multidimensional relationships through rapid cohort construction and hypothesis generation, while maintaining conventional statistical and experimental validation as essential components of inference. Future studies incorporating diverse populations, detailed treatment histories, genomic ancestry, longitudinal sampling, and functional validation will be critical for determining whether context-specific RTK-RAS patterns can ultimately improve risk stratification and therapeutic selection in EOCRC.

## Introduction

Early-onset colorectal cancer (EOCRC), conventionally defined as colorectal cancer (CRC) diagnosed before 50 years of age, has emerged as one of the most concerning epidemiologic trends in contemporary oncology. Although improvements in screening and prevention have contributed to declining CRC incidence among many older populations, the incidence of disease among younger adults continues to increase. Importantly, EOCRC should not be regarded simply as conventional CRC occurring several decades earlier. Increasing molecular, epidemiologic, microbiome, and clinical evidence suggests that tumors arising in younger patients may develop under distinct combinations of inherited susceptibility, environmental exposures, microbial influences, molecular evolution, and host biology [1–5]. Recent whole-genome analyses have further demonstrated age-related differences in colorectal carcinogenesis, including differences in mutational signatures that may reflect exposures occurring much earlier in life.

Within this evolving landscape, Diaz and colleagues recently investigated the receptor tyrosine kinase-RAS (RTK-RAS) pathway across 2,515 CRC cases stratified simultaneously by age at diagnosis, Hispanic/Latino (H/L) versus non-Hispanic White (NHW) classification, and exposure to FOLFOX chemotherapy [6]. Rather than identifying a universally adverse or favorable effect of RTK-RAS alterations, the study revealed a more nuanced pattern: the clinical implications of pathway alterations differed according to the biological and therapeutic context in which they occurred. Most notably, RTK-RAS alterations were associated with worse overall survival among untreated EOCRC NHW patients but with improved survival among FOLFOX-treated late-onset NHW patients [6]. At the same time, overall pathway alteration frequencies remained relatively stable across many of the studied groups, whereas individual RTK-RAS genes displayed substantially greater heterogeneity.

These observations raise a broader issue with relevance well beyond this particular pathway. Precision oncology has traditionally sought biomarkers that can classify tumors into relatively stable categories, mutated versus wild type, amplified versus non-amplified, or biomarker-positive versus biomarker-negative. The findings of Diaz et al. instead support a model in which the meaning of a genomic alteration can be conditional. Its prognostic or therapeutic relevance may depend on age at disease development, treatment history, molecular background, ancestry-related factors, and potentially the tumor microenvironment. This conceptual transition, from static biomarkers to context-dependent molecular states, may be particularly important for understanding EOCRC.

The novelty of this Commentary lies in extending these empirical findings into a broader framework for context-aware biomarker interpretation. Whereas Diaz et al. identified subgroup-specific RTK-RAS patterns and contrasting survival associations, we propose that a biomarker should not be regarded solely as a fixed, binary tumor characteristic. Instead, its clinical meaning may emerge from interactions among the specific molecular alteration, age-related tumor biology, treatment exposure, ancestry-related factors, and the wider genomic and microenvironmental state of the tumor. This Commentary therefore moves beyond summarizing the original study by defining the concept of a context-conditioned molecular state and outlining the analytical and experimental strategies required to distinguish prognostic associations from treatment-predictive effects and ultimately establish clinical utility.

Importantly, these findings require an early distinction between prognostic and predictive biomarkers. A prognostic biomarker is associated with clinical outcome independently of a specific therapeutic intervention, whereas a predictive biomarker identifies differential benefit from one treatment relative to another. The subgroup-specific survival associations reported by Diaz et al. may indicate context-dependent prognostic relationships; however, comparisons of associations within FOLFOX-exposed and non-exposed groups do not, by themselves, establish that RTK–RAS alteration status predicts benefit from FOLFOX. Demonstration of predictive utility would require formal treatment-by-biomarker interaction analyses, rigorous adjustment for treatment allocation and other potential confounders, and validation in independent, appropriately designed cohorts. Accordingly, the findings discussed throughout this manuscript should be interpreted as hypothesis-generating associations rather than evidence supporting treatment selection.

## RTK-RAS Alterations: From Pathway Prevalence to Biological Context

The RTK-RAS signaling network is central to colorectal carcinogenesis and interacts with multiple oncogenic pathways controlling tumor initiation, progression, and therapeutic response [7]. Signaling initiated by receptor tyrosine kinases converges on RAS, RAF, and downstream MAPK effectors to regulate proliferation, survival, differentiation, metabolism, and interactions with the tumor microenvironment. Canonical alterations involving KRAS, NRAS, and BRAF have long-established clinical relevance, particularly for selection of anti-EGFR therapy in metastatic CRC. However, viewing the RTK-RAS pathway exclusively through these major drivers may obscure substantial biological diversity created by less common alterations in receptors, negative regulators, and downstream signaling components.

This point is illustrated by the focal study. Approximately two-thirds or more of tumors in each major age- and ancestry-defined subgroup contained at least one RTK-RAS alteration, yet aggregate pathway prevalence did not differ significantly by FOLFOX exposure. The biologically interesting differences became apparent only after pathway-level information was decomposed into individual genes and clinically defined strata [6]. In EOCRC H/L tumors, for example, ERBB2, NF1, and FGFR2 alterations were more common in the non-FOLFOX group, whereas NTRK2 alterations were enriched among untreated late-onset H/L cases. Among untreated EOCRC patients, ERBB2, MAPK3, CBL, and NF1 were more frequent in H/L than NHW tumors. These observations demonstrate why two tumors classified simply as “RTK-RAS altered” may represent markedly different biological states.

Large-scale sequencing studies increasingly support this more granular interpretation of CRC biology. Whole-genome characterization of more than 2,000 CRCs has revealed extensive heterogeneity involving driver events, mutational processes, structural variation, and molecular subgroups, with age-associated molecular differences that are not captured by individual canonical drivers alone [8]. This complexity is especially pertinent to EOCRC, where the mechanisms responsible for the rising incidence remain incompletely resolved.

For this analysis, an RTK–RAS-altered tumor was defined as a tumor harboring at least one protein-altering mutation in a gene assigned to the RTK–RAS pathway in the source dataset, including KRAS, NRAS, BRAF, ERBB2, NF1, FGFR2, NTRK2, CBL, and MAPK3. This aggregate classification was used as a pathway-level organizational framework and should not be interpreted as defining a biologically homogeneous biomarker category. The included genes occupy different positions within the signaling network and may have distinct mechanisms and consequences. For example, KRAS, NRAS, and BRAF are canonical CRC drivers with established clinical relevance, whereas alterations involving receptor tyrosine kinases, negative regulators such as NF1 and CBL, and downstream effectors such as MAPK3 are less frequent and may vary in oncogenic strength, functional significance, and clinical actionability. Moreover, the presence of a genomic alteration does not necessarily establish functional activation of the pathway. We therefore interpret the aggregate RTK-RAS classification as hypothesis-generating and emphasize gene-specific findings wherever possible. Future studies should evaluate individual alteration types, including activating mutations, loss-of-function events, copy-number changes, and gene fusions, and integrate transcriptomic and proteomic measurements to determine their functional effects within the broader molecular and treatment context.

Importantly, the presence of a genomic alteration in an RTK-RAS pathway gene should not be interpreted as direct evidence of functional pathway activation. The mutation-based classification used in this study identifies tumors harboring alterations in pathway components but does not measure downstream signaling activity or establish the functional consequences of individual variants. RTK-RAS activation is additionally influenced by gene expression, copy-number changes, protein abundance and phosphorylation, ligand availability, feedback regulation, cellular composition, and interactions among malignant, stromal, and immune cells within the tumor microenvironment. Conversely, pathway activation may occur in the absence of detectable mutations through non-genomic regulatory mechanisms. Therefore, the associations reported here reflect RTK-RAS genomic alteration status, rather than confirmed pathway activation, and should be interpreted as hypothesis-generating. Future studies integrating transcriptomics, proteomics, and spatial profiling, together with functional validation in organoid or patient-derived experimental models, will be necessary to determine whether the identified genomic alterations produce biologically meaningful RTK-RAS activation and influence treatment response.

Because multiple RTK-RAS genes were evaluated across several age-, ancestry-, and treatment-defined subgroups, the gene-level analyses are subject to an increased risk of false-positive findings resulting from multiple comparisons. In particular, nominal associations involving ERBB2, NF1, FGFR2, NTRK2, MAPK3, and CBL should be interpreted cautiously, especially given the relatively small sizes of some subgroups and the low frequencies of several alterations. These findings are therefore exploratory and hypothesis-generating rather than confirmatory. Future studies should prespecify primary comparisons, apply appropriate multiple-testing procedures, such as false discovery rate control, and validate the observed associations in larger, independent, and ancestrally diverse cohorts. Until such validation is completed, these gene-specific differences should not be interpreted as established biomarkers or used to guide clinical decision-making.

Consequently, pathway-level classification may be most informative when considered as an organizational framework rather than as a single biomarker. This concept is consistent with evidence from consensus molecular subtype analyses demonstrating that broader molecular states of CRC can provide clinically relevant prognostic information and influence associations with treatment outcomes [9]. The relevant question may therefore no longer be simply whether RTK-RAS is altered, but which component is altered, in what genomic background, at what age the tumor developed, and under what therapeutic exposure the outcome is being measured.

## Prognostic Polarity and the Importance of Treatment Context

Perhaps the most provocative observation from the focal study is the context-dependent direction of the association between RTK–RAS alterations and overall survival. Among untreated NHW patients with EOCRC, RTK–RAS alterations were associated with inferior overall survival, whereas among FOLFOX-treated NHW patients with late-onset disease, pathway alterations were associated with improved survival [6]. These findings demonstrate prognostic heterogeneity across clinically defined subgroups but should not be interpreted as evidence of predictive utility. In particular, an association observed within a treated subgroup does not establish that biomarker-positive patients derive greater benefit from that treatment than biomarker-negative patients.

Several biological explanations are possible. In untreated EOCRC, RTK-RAS dysregulation may identify tumors with greater proliferative capacity, adaptive signaling, metastatic potential, or cooperation with additional adverse genomic events. Conversely, exposure to fluoropyrimidine and oxaliplatin could alter the relationship between pathway state and outcome through effects on DNA damage, replication stress, cell-cycle control, clonal selection, or treatment sensitivity. Age-associated differences in tumor evolution, immune contexture, stromal interactions, DNA repair, and competing molecular pathways could further modify these relationships. Clinical evidence also suggests that age at diagnosis may influence the therapeutic context in which oxaliplatin-based chemotherapy is administered. Analyses from the IDEA database comparing treatment adherence, toxicity, and outcomes following three versus six months of adjuvant fluoropyrimidine and oxaliplatin provide an important clinical framework for interpreting chemotherapy outcomes specifically in EOCRC [10].

Importantly, however, observational treatment comparisons cannot establish that FOLFOX itself caused these differences. Patients receiving FOLFOX differ from untreated patients for numerous reasons, including stage, resectability, comorbidities, performance status, treatment era, metastatic burden, treatment sequencing, and clinician or patient preferences. The original analysis appropriately identifies treatment-selection bias and residual confounding as important limitations. Therefore, the survival associations should currently be interpreted as hypothesis-generating rather than as evidence that RTK-RAS alteration status predicts benefit from FOLFOX.

The interpretation of these context-dependent associations also requires careful consideration of clinicopathologic and treatment-related confounding factors beyond age, ancestry, and documented FOLFOX exposure. Tumor stage, sidedness and anatomical location, MSI/MMR status, BRAF status, metastatic involvement and disease burden, performance status, and comorbidities may each be associated with tumor biology, treatment selection, and survival. For example, MSI/MMR status and BRAF alterations define clinically and biologically distinct CRC subgroups that may differ in prognosis, anatomical distribution, immune characteristics, and therapeutic response. Similarly, patients with localized, resectable disease may receive FOLFOX in an adjuvant setting, whereas those with metastatic disease may receive it as part of a more complex treatment sequence. Subsequent systemic therapies, including anti-EGFR- or anti-VEGF-based treatment, immunotherapy, and later-line regimens, could further modify overall survival. Differences in these variables across the analyzed subgroups could therefore confound or partially explain the apparent prognostic polarity of RTK-RAS alterations. In the absence of complete adjustment for these factors, the reported associations should not be interpreted as demonstrating an independent prognostic effect of RTK-RAS status or a predictive benefit from FOLFOX.

Nevertheless, newer evidence provides an intriguing external context. A 2026 study evaluating stage III and high-risk stage II CRC reported a significant interaction between KRAS/BRAF status and adjuvant chemotherapy regimen. Oxaliplatin-containing chemotherapy was associated with improved survival in patients with KRAS-mutated tumors compared with fluoropyrimidine monotherapy, whereas patients with BRAF-mutated cancers showed a different association [11]. Although the population, design, and biomarker definitions differ from those of Diaz et al., the study independently reinforces an important principle: the relationship between RAS-RAF biology and chemotherapy outcomes may depend on the specific molecular alteration and treatment context rather than following one universal direction.

Consistent with the distinction introduced earlier, the current findings support context-dependent prognostic associations but do not establish RTK–RAS status as a predictive biomarker of FOLFOX benefit. Kaplan–Meier analyses conducted separately within treatment groups can identify clinically relevant outcome associations, but predictive utility requires a formal comparison of treatment effects according to biomarker status. Future validation should therefore incorporate multivariable Cox models, propensity-based methods where appropriate, and explicit treatment-by-biomarker interaction tests, ideally followed by confirmation in independent prospective cohorts.

## EOCRC May Carry Molecular Imprints of Earlier-Life Exposures

An additional development since the emergence of this work is growing evidence that EOCRC may contain molecular traces of exposures occurring years or decades before diagnosis. A multinational whole-genome study of 981 CRCs from 11 countries reported age- and geography-associated differences in mutational processes. Particularly noteworthy was enrichment of the colibactin-associated SBS88 and ID18 signatures in cancers diagnosed at younger ages; these signatures were substantially more common in individuals diagnosed before age 40 than in older patients and appeared to originate early during tumor development [12].

This observation broadens the framework through which RTK-RAS differences should be interpreted. Age-specific pathway alterations may not necessarily arise from age itself. Rather, age at diagnosis may function as a surrogate for different carcinogenic trajectories involving microbial exposures, diet, obesity and metabolic factors, inflammatory states, environmental exposures, inherited susceptibility, or combinations of these factors.

Future analyses could therefore connect RTK-RAS states with mutational signatures, microbiome composition, methylation, transcriptomic programs, and spatial characteristics of the tumor microenvironment. Such integration could determine whether alterations such as NF1 or ERBB2 enrichment in particular EOCRC populations represent isolated genomic observations or components of broader molecular phenotypes.

The increasing availability of single-cell and spatial technologies makes this particularly relevant. RTK-RAS signaling cannot be completely understood from DNA sequence alone because pathway activity is influenced by gene expression, protein phosphorylation, feedback regulation, cellular composition, ligand availability, and interactions between malignant, stromal, and immune compartments. Thus, a tumor containing an RTK-RAS mutation is not necessarily equivalent to a tumor with functional activation of the pathway. Multi-omic and spatial validation represents a logical next step for determining the biological consequences of the subgroup-specific genomic associations identified in the original study.

## Ancestry-Aware Precision Oncology Without Biological Reductionism

The ancestry-associated observations deserve careful interpretation. In the focal analysis, several gene-level differences were observed among untreated EOCRC patients, including higher frequencies of ERBB2, MAPK3, CBL, and NF1 alterations among H/L compared with NHW patients [6]. However, aggregate RTK-RAS alteration frequencies were similar between groups, and no corresponding survival association was identified among H/L patients.

Importantly, self-reported race and ethnicity, genetic ancestry, and socioeconomic or environmental determinants represent related but conceptually distinct dimensions. Self-reported race and ethnicity primarily reflect social identity, culture, lived experience, and administrative classification and should not be interpreted as direct measures of genetic ancestry. Genetic ancestry instead reflects inherited genomic variation and can be estimated using ancestry-informative genetic markers. Socioeconomic conditions, environmental exposures, neighborhood context, healthcare access, comorbidities, and treatment patterns may correlate with racial or ethnic classifications but are not interchangeable with either self-reported identity or genetic ancestry. Whenever possible, these variables should be measured directly rather than inferred from race or ethnicity.

This distinction is particularly important for H/L populations, which are highly heterogeneous and frequently admixed, with varying proportions of Indigenous American, European, African, and other ancestries. Future studies should therefore complement self-reported race and ethnicity with estimates of global and, where biologically relevant, local genetic ancestry. Such analyses could help determine whether observed mutation-frequency differences correlate with inherited genomic ancestry or are more strongly associated with environmental, socioeconomic, clinical, or sampling factors. They could also reduce potential misclassification arising from administrative or surname-derived annotations, as acknowledged in the original report.

The sample sizes of the H/L subgroups also warrant caution. Stratification by age at diagnosis and FOLFOX exposure produced relatively small analytical groups, limiting statistical power for robust gene-level comparisons, particularly for genes altered at low frequencies. The reported differences involving ERBB2, MAPK3, CBL, NF1, FGFR2, and NTRK2 should therefore be regarded as exploratory and hypothesis-generating rather than definitive ancestry-associated molecular differences. Multiple subgroup and gene-level comparisons may further increase the probability of chance findings. Validation will require larger, independent H/L cohorts with sufficient representation of diverse backgrounds, detailed subgroup information, genomic ancestry estimates, standardized molecular profiling, and appropriate control for multiple testing and relevant clinical covariates.

More broadly, equitable precision oncology requires more than including race or ethnicity as a covariate in a statistical model. It requires sufficiently large and deeply characterized cohorts from historically underrepresented populations, harmonized biospecimen collection, consistent molecular platforms, granular treatment information, and direct integration of social and environmental determinants with tumor biology. These approaches will be necessary to distinguish potentially ancestry-associated molecular signals from differences related to healthcare access, exposure patterns, treatment, sampling, or other sources of confounding.

## Artificial Intelligence as a Discovery Layer, not a Replacement for Inference

A second major contribution of the focal article is methodological. AI-HOPE and the pathway-specialized AI-HOPE-RTK-RAS agent were used to interrogate integrated genomic, clinical, and treatment information through natural-language queries, rapidly defining cohorts and identifying candidate associations for subsequent statistical testing [6]. The importance of this approach lies less in automating a particular statistical test than in reducing the practical barrier to exploring high-dimensional clinical-genomic hypotheses.

Traditional bioinformatics workflows can become cumbersome when investigators repeatedly need to ask questions involving combinations of age, ancestry, treatment, survival, tumor subtype, pathway status, and individual genomic alterations. Conversational AI and agent-based systems potentially create an interface between human biological reasoning and computationally structured datasets. In this model, an investigator may formulate a clinical question directly, while specialized agents translate the question into defined cohorts, retrieve relevant variables, generate comparisons, and return interpretable outputs.

This architecture becomes increasingly valuable as precision oncology moves from genomics toward multimodal datasets incorporating transcriptomics, proteomics, epigenomics, microbiome profiles, imaging, spatial data, electronic health records, and social determinants of health. Contemporary AI research is likewise moving toward models that integrate heterogeneous biomedical modalities rather than analyzing each data layer independently.

Yet the distinction between AI-assisted discovery and statistical evidence is essential. AI should facilitate cohort construction, exploration, prioritization, and visualization; it should not confer validity on an association merely because that association was identified efficiently. The focal study follows the appropriate principle by subjecting AI-derived hypotheses to conventional statistical evaluation.

The next generation of systems should strengthen this framework further through auditable query histories, predefined statistical analysis plans for confirmatory testing, automatic detection of small subgroups, multiple-testing correction, confounder assessment, provenance tracking, reproducible code generation, and independent validation datasets. These safeguards are particularly important because conversational AI can make complex analyses appear deceptively simple. Ease of querying should increase scientific accessibility without lowering the evidentiary threshold required for biological or clinical conclusions.

The potential is nevertheless substantial. A 2025 multicenter EOCRC investigation demonstrated that machine-learning approaches integrating tumor-derived biomarkers could stratify recurrence and overall-survival risk and subsequently validate those predictions in independent patients [13]. Such work illustrates the direction in which AI-enabled EOCRC research can progress: from efficient retrospective discovery toward externally validated models capable of informing clinically meaningful risk stratification.

## From Exploratory Associations to Clinically Actionable Biomarkers

The observations described by Diaz et al. generate several testable hypotheses rather than immediate changes in treatment. Their greatest value may therefore lie in defining the next generation of studies needed to move RTK-RAS profiling toward clinical utility.

Independent validation should first determine whether the survival associations persist in larger H/L and other ancestrally diverse populations. Future cohorts should include detailed information about stage, primary tumor location, metastatic sites, MSI status, sidedness, surgery, FOLFOX timing, number of cycles, dose intensity, subsequent systemic treatments, anti-EGFR or anti-VEGF exposure, and cause-specific outcomes. Formal multivariable modeling would determine whether pathway status provides prognostic information beyond established clinicopathologic factors. Importantly, RTK-RAS alterations should ultimately be evaluated alongside other emerging molecular predictors of chemotherapy outcome. For example, microRNA-based signatures have demonstrated potential for both risk stratification and prediction of response to FOLFOX-based adjuvant therapy in stage II-III CRC [14], illustrating how complementary molecular layers could potentially be integrated with pathway-level genomic information.

The genomic definition of RTK-RAS should also expand beyond protein-altering mutations. Copy-number gains involving genes such as ERBB2 or MET, clinically relevant gene fusions, transcriptomic activation, phosphoproteomic signaling, and downstream pathway activity may identify tumors missed by mutation-only classification. Longitudinal sampling and circulating tumor DNA could additionally establish whether RTK-RAS clones change during FOLFOX exposure and whether clonal dynamics correlate with therapeutic response. The rapidly expanding use of blood- and stool-based biomarkers for CRC detection, surveillance, and disease monitoring provides a complementary framework for translating these molecular observations into minimally invasive longitudinal strategies [15].

Although opposite survival associations were observed across the FOLFOX-exposed and non-exposed subgroups, these findings should not be interpreted as evidence that FOLFOX biologically modifies the effect of RTK–RAS alterations. Because treatment exposure was not randomly assigned, the observed patterns may reflect treatment-selection bias, differences in disease stage or clinical characteristics, treatment sequencing, molecular background, and residual or unmeasured confounding. Moreover, Kaplan-Meier analyses conducted separately within treatment-defined subgroups do not establish a formal treatment-by-biomarker interaction or demonstrate that RTK–RAS alteration status predicts benefit from FOLFOX. Therefore, these results represent treatment-associated, hypothesis-generating survival patterns rather than evidence of a causal biological treatment effect. Establishing predictive or effect-modifying utility will require formal interaction analyses in adequately powered cohorts, comprehensive adjustment for treatment allocation and clinical covariates, independent validation, and ideally prospective or experimental confirmation.

The RTK-RAS patterns discussed here should be regarded as exploratory, hypothesis-generating associations rather than validated prognostic, predictive, or clinically actionable biomarkers. Although these findings may help prioritize molecular hypotheses and guide the design of future studies, they should not currently be used for therapeutic selection or clinical decision-making. Establishing clinical utility will require independent external replication, adequately powered prospective studies, adjustment for relevant clinicopathologic and treatment-related confounders, formal treatment-by-biomarker interaction testing, and functional validation. Accordingly, the present framework represents a discovery-oriented application of precision-oncology research that may support future biomarker development but does not establish immediate clinical actionability.

Finally, biological validation is essential. Organoids, patient-derived models, and experimental systems representing early- and late-onset CRC could test whether specific alterations such as NF1 loss or ERBB2 activation modify sensitivity to fluoropyrimidine or oxaliplatin. Such models could also assess whether the effect depends on additional mutations, microsatellite status, cellular lineage, or microenvironmental context.

## Conclusions

The principal lesson emerging from RTK-RAS analysis in EOCRC is not that pathway alterations are universally favorable or unfavorable. Rather, their meaning appears to depend on context. Age at cancer onset, treatment exposure, ancestry-related factors, specific pathway components, and the wider molecular state of the tumor may collectively determine whether an alteration is biologically aggressive, therapeutically relevant, clinically neutral, or simply a marker of another underlying process.

Recent genomic and treatment-outcome studies strengthen this conceptual framework. Whole-genome analyses demonstrate that colorectal cancers developing at younger ages can harbor distinct mutational processes, while emerging evidence indicates that even canonical KRAS and BRAF alterations can interact differently with oxaliplatin-based treatment. The future of CRC precision oncology may therefore require moving beyond static single-gene or pathway-positive classifications toward models that integrate who the patient is, when the cancer developed, how the tumor evolved, and what therapeutic pressures it encountered.

AI-enabled frameworks such as AI-HOPE provide a potentially powerful mechanism for navigating this multidimensional space. Their most valuable role is not to replace conventional biostatistics, experimental validation, or clinical judgment, but to connect these disciplines more efficiently by transforming complex datasets into testable hypotheses. If coupled with prospective validation, ancestrally diverse cohorts, longitudinal treatment information, and multi-omic characterization, this strategy could help transform increasingly complex molecular data into more precise and equitable approaches to EOCRC management.

The conceptual advance of this Commentary is the proposal that precision-oncology biomarkers should be evaluated as dynamic, context-conditioned molecular states rather than as universally interpretable binary variables. Building upon the subgroup-specific observations of Diaz et al., this framework integrates the identity of the alteration with age at disease onset, treatment exposure, ancestry-related factors, tumor evolution, and the broader multi-omic and microenvironmental landscape. It also provides a translational roadmap for evaluating such biomarkers through formal treatment-by-biomarker interaction testing, diverse and independently validated cohorts, longitudinal sampling, functional modeling, and multi-omic and spatial analyses. This context-aware approach may improve the biological interpretation, clinical validation, and equitable application of biomarkers in EOCRC and, more broadly, across precision oncology.
